# Circulating Extracellular Vesicles Reflect Dynamic Shifts in Liver Transcriptome Following Tumour Resection

**DOI:** 10.3390/cancers18132109

**Published:** 2026-06-29

**Authors:** Lauren A. Newman, Daniel Daly, Fiona Whelan, Janina Kaczmarczyk, Eu Ling Neo, John W. Chen, Mark E. Brooke-Smith, Andrew Rowland, Sonja Klebe, Savio George Barreto, Zivile Useckaite

**Affiliations:** 1 Flinders Health and Medical Research Institute, College of Medicine and Public Health, Flinders University, Adelaide, SA 5042, Australia; 2 HPB and Liver Transplant Unit, Flinders Medical Centre, Bedford Park, SA 5042, Australia; 3 Department of Molecular and Biomedical Science, University of Adelaide, Adelaide, SA 5005, Australia; 4 Adelaide Microscopy, University of Adelaide, Adelaide, SA 5005, Australia; 5 Department of Surgical Pathology, SA Pathology at Flinders Medical Centre, Adelaide, SA 5042, Australia

**Keywords:** extracellular vesicles, liquid biopsy, blood biomarkers, molecular oncology, hepatocellular carcinoma

## Abstract

**Background/Objectives**: Poor outcomes in liver cancer are often driven by late-stage diagnoses and high recurrence rates following surgical resection, highlighting a critical clinical need for non-invasive surveillance tools. This proof-of-concept study investigates the utility of circulating extracellular vesicles (EVs) to track dynamic molecular shifts and monitor patient response following tumour resection. **Methods**: Small ribonucleic acid (RNA) sequencing was conducted on matched tumour tissue, tissue-derived EVs, and plasma EVs collected at the time of surgery from patients with liver cancer. To capture longitudinal transcriptomic changes, plasma EVs were also collected at a post-operative follow-up appointment. **Results**: At the time of surgery, the transcriptomic profile of circulating plasma EVs strongly correlated with both the matched tumour tissue and tissue-derived EVs, exhibiting substantial transcript overlap. However, at post-operative follow-up, the circulating EV cargo significantly diverged from the primary tumour profile. This loss of similarity was characterised by a distinct shift in RNA cargo, including 173 uniquely detected transcripts absent at baseline. **Conclusions**: Circulating EVs accurately reflect the local hepatic transcriptome at the time of surgery, but their profile dynamically and fundamentally diverges once the tumour is removed. This post-surgical divergence provides an initial proof-of-concept that utilising patients as their own internal control to longitudinally profile EV cargo may track the clearance of tumour signals and monitor post-surgical systemic changes, highlighting their potential utility for future longitudinal studies aimed at tracking cancer progression and recurrence.

## Simple Summary

Liver cancer recurrence remains a major challenge following surgical resection. Currently, sensitive, non-invasive tools to detect minimal residual disease are lacking. This study investigated circulating extracellular vesicles (EVs)—particles that shuttle molecular cargo between cells—as a dynamic liquid biopsy. Sequencing the small RNA content in tumour tissue and matched plasma EVs suggested that circulating EVs at the time of surgery closely mirror the tumour’s transcriptomic profile. In blood collected weeks after tumour removal, this EV RNA cargo diverged from the baseline tumour signature, which may reflect the clearance of tumour-derived signals and the body’s post-surgical physiological response. This proof-of-concept study highlights that longitudinal profiling of circulating EVs could capture real-time molecular adaptations, with the potential to be used as a minimally invasive strategy for treatment monitoring.

## 1. Introduction

Liver cancer represents a significant global health burden, as the sixth most diagnosed cancer, and third leading cause of cancer-related mortality. The annual incidence is projected to grow by more than 55%, reaching an estimated 1.4 million new cases and 1.3 million deaths worldwide by 2040 [1]. Hepatocellular Carcinoma (HCC), the most common primary liver cancer [2], often develops in the setting of chronic liver disease, including hepatitis B and C infections, cirrhosis, and Metabolic Associated Fatty Liver Disease (MAFLD) [3,4]. The development and progression of HCC is complex and heterogenous, driven by genetic alterations, epigenetic dysregulation, and multiple tumour–stromal interactions within the hepatic microenvironment [5,6].

Poor outcomes are driven by late-stage diagnoses, which impede the effectiveness of curative treatment options [7]. The lack of sensitive, non-invasive diagnostic tools for early detection, coupled with the histological and molecular heterogeneity of HCC, pose a major clinical challenge [8]. The liver is also a common site for metastasis, which affects 20–30% of patients with Colorectal Cancer (CRC) [9,10,11]. Detailed pathological and molecular analysis of metastases is essential for selecting appropriate treatment strategies [12,13,14,15], and for furthering the understanding of the biological behaviour of these tumours.

For patients with many primary or secondary liver cancers, surgical resection offers the best chance for long-term survival [16]. However, tumour recurrence remains a major challenge, particularly in HCC patients who have underlying cirrhosis or persistent metabolic risk factors [16]. Accordingly, effective post-surgical surveillance is important, but is currently limited in capability to detect minimal residual disease or early signs of recurrence [17]. To address this important clinical need, liquid biopsy approaches including circulating extracellular vesicles (EVs) have attracted significant attention as a minimally invasive tool for real-time tumour monitoring. EVs are lipid bilayer-enclosed particles of various size and molecular composition, loaded with proteins, nucleic acids, lipids, and metabolites inherited from their releasing cells [4]. Tumour-derived EVs have been shown to play an active role in cancer progression by mediating intercellular communication, modulating the immune response, and remodelling the tumour microenvironment [18]. EVs are also increasingly recognised as key mediators of systemic responses, facilitating communication between injured tissues and remote organs [19].

The shuttling of small non-coding ribonucleic acids (sncRNAs) between cells is one mechanism by which biological pathways may be modulated by EVs [20]. Thus, profiling the EV transcriptomic cargo before and after surgery offers the opportunity to capture dynamic changes in the tissue phenotype and inter-organ cross-talk, reflecting molecular signals of tissue repair or monitoring recurrence risk. However, the extent to which the transcriptomic content of circulating EVs reflects that of the primary tumour and EVs within the local tissue environment remains poorly understood. In this study, transcriptomic profiling was performed on tumour tissue, tissue-derived EVs, and plasma EVs collected at the time of surgery and post-operative follow-up, with the goal to characterise the shared and unique small RNA signatures across these compartments and explore the utility of circulating EVs to signal changes in liver biology in HCC.

## 2. Materials and Methods

### 2.1. Recruitment

Patients identified as requiring liver surgery by the Hepatopancreatobiliary multidisciplinary team at Flinders Medical Centre were identified for enrolment into the study. Patient demographics are outlined in Table 1. The study protocol was approved by the Southern Adelaide Clinical Human Research Ethics Committee (LNR/23/SAC/65), and written informed consent was obtained from each participant prior to study enrolment. The study was conducted according to the principles stated in the Declaration of Helsinki and was compliant with CPMP/ICH/135/95 GCP standards.

### 2.2. Sample Collection and Processing

Venous blood was collected on the day of surgery and at patient’s follow-up appointment (3–7 weeks following surgery). Eight millilitres of whole blood was collected into Greiner K2 EDTA Tubes (Greiner Bio-One, Frickenhausen, Germany). Plasma was isolated from whole blood within 30 min of sample collection by two cycles of centrifugation at 2500× *g* for 15 min at 4 °C. Plasma samples were aliquoted and stored at −80 °C until required for analysis.

Resected liver tissue (2 g approx.) containing tumour and non-tumour tissue was placed in cryogenic vials within 30 min of resection, and vials were sealed and frozen in liquid nitrogen (LN2) vapour before submerging in LN2 for two minutes. Specimens were stored at −80 °C until required for analysis.

### 2.3. Histochemistry

Full-face histopathology slides were prepared from frozen tissue samples and stained with H&E stain. Tissue morphology was assessed and full-face histopathology slide for each tissue sample was used to guide the cutting of the tissue sample into two equal parts (assuring that tissue used for homogenisation is matched as closely as possible to the tissue sample used for EV isolation) (Supplementary Figure S1).

### 2.4. Cryo-Sectioning

Unfixed frozen tissue samples (up to 2 cm in diameter) were imbedded into tissue moulds using two drops of Tissue-Tek optimal cutting temperature (OCT) medium (Sakura Finetek, Torrance, CA, USA). Full-face tissue sections (10 µm thickness) were cut using a cryostat at −20 °C. Within 1 min of cutting, tissue sections were transferred onto Superfrost Plus microscope slides (Fisher Scientific, Waltham, MA, USA). Tissue sections were allowed to melt onto the slide and to dry at room temperature (RT) before fixing them in formalin.

### 2.5. Haematoxylin and Eosin Staining (H&E)

Following cryo-sectioning, full-face tissue sections were fixed onto Superfrost Plus microscope slides (Fisher Scientific, Waltham, MA, USA) by placing slides in formalin for 10 min at RT, followed by a wash in deionized water for 2 min. Tissue sections were stained with haematoxylin for 5 min, followed by a wash in tap water for 1 min. Excess haematoxylin stain was removed by dipping the slides in acid alcohol and washing with tap water for 1 min. The tissues were blued by incubating the slides in Lithium Carbonate for 4 min, followed by a dip in deionized water. Haematoxylin-stained slides were incubated in Eosin for 2 min to allow counterstaining of extracellular matrix and cytoplasm. The slides were washed in alcohol and dehydrated in Xylene for 5 min. The slides were mounted by applying a small amount of mounting medium onto the tissue and slowly lowering the coverslip. The slides were allowed to dry in a horizontal position.

### 2.6. Whole Slide Imaging

The histopathology slides were imaged using Olympus Brightfield BX53 Upright Microscope (Olympus Corporation, Tokyo, Japan), Mag. 40×.

### 2.7. Human Liver Tissue Homogenisation

Liver tissue samples (0.1 g) were placed in polycarbonate centrifuge tubes containing 3 mL ice-cold Phosphate KCl Buffer solution (10 mM phosphate buffer, pH = 7.4 with 1.15% *w*/*v* potassium chloride). The tissue samples were minced using surgical scissors and homogenised using the Ultra Turrax T25 (IKA, Staufen, Germany) set at 20,500 rpm, allowing 2 × 30 s strokes with 30 s cooling period between bursts. Ground liver and intestinal tissue samples were transferred into an ice-cold Potter-Elvehjam homogeniser (Heidolph, Schwabach, Germany) and homogenised using an electric drill, allowing eight full strokes with the drill at full speed. The resulting tissue homogenates were aliquoted in protein LoBind tubes (Eppendorf, Hamburg, Germany) and stored at −80 °C until required for analysis.

### 2.8. Tissue Preparation for EV Isolation

Liver tissue (0.1 g) was sliced into 2 × 2 mm sections (approx.) by using a disposable scalpel and transferred into 2 mL Protein LoBind tubes (Eppendorf, Hamburg, Germany). The tissue slices were incubated in 1 mL Roswell Park Memorial Institute (RPMI) 1640 medium supplemented with 2 mg/mL of collagenase D and 40 U/mL DNase I, for 45 min at 37 °C in water bath, shaking gently.

Following incubation, RPMI-1640 medium containing tissue was strained using 40 µM nylon cell strainer (BD Falcon, Franklin Lakes, NJ, USA), allowing tissue-conditioned media (TCM) to pass through the filter by gravity. An additional 150 µL of freshly filtered (0.2 µm) phosphate-buffered saline (PBS) was added and allowed to drain through the filter by gravity, into the tube containing TCM. The resulting TCM was centrifuged at 300× *g* for 10 min at 4 °C, and the supernatant was transferred to a new tube and centrifuged at 2000× *g* for 20 min at 4 °C. The resulting TCM was used for EV isolation by size exclusion chromatography (SEC) using 1 mL Cytiva CL2B SEC columns (Cytiva, Malborough, MA, USA).

### 2.9. Size Exclusion Chromatography (SEC)

Briefly, prior to EV isolation, SEC columns were conditioned by washing with 20 mL of freshly filtered (0.2 µm) PBS. Tissue-conditioned media (1 mL) or plasma (1 mL) was added to the sample reservoir and EVs were eluted in PBS, which was added to the sample reservoir as the last of the sample entered the column. For the duration of the EV isolation, the volume of PBS in the reservoir was kept below 2 mL. The initial five fractions (2.5 mL/0.5 mL per fraction) of flow-through were discarded, and EVs were collected as pooled fractions 6 to 9 (2 mL total) using 5 mL Protein LoBind tubes (Eppendorf, Hamburg, Germany). The resulting pooled EV fractions were mixed by gentle inversion 8 to 10 times and concentrated using preconditioned Amicon Ultra 4 (30 KDa) centrifuge filters (MilliporeSigma, Burlington, MA, USA) to a final volume of 400 µL or 200 µL in PBS for tissue or plasma source, respectively. The samples were then aliquoted (to avoid freeze-thaw) and stored at −80 °C until required for analysis.

### 2.10. Nanoparticle Tracking Analysis (NTA)

Nanoparticle tracking analysis (NTA) was performed to determine global particle abundance and size distribution using the NanoSight NS300 (Malvern Panalytical, Malvern, UK, Software Version 3.4). Samples were diluted between 1:2000 and 1:10,000 using freshly 0.2 µm filtered PBS; five 60 s videos were captured and analysed under constant flow conditions (flow rate 50) using NTA 3.4 software.

### 2.11. Transmission Electron Microscopy (TEM)

Liver tissue sections were prepared and imaged using a previously published protocol [21]. Small pieces (1mm^3^) of liver tissue were immersed in primary fixative (2.5% glutaraldehyde/4% Paraformaldehyde in PBS with 4% sucrose) for 48h at 4 °C, then washed in PBS and 4% sucrose. The tissue was then immersed in the secondary fixative (aqueous 2% osmium tetroxide) for 1 h, followed by dehydration through a graded series of ethanol solutions (50−100%) and 100% propylene oxide. The tissue was then infiltrated with 1:1 and 2:1 mixtures of epoxy resin/propylene oxide followed by three changes of 100% epoxy resin. The tissue was embedded in fresh resin in BEEM capsules and polymerized in the oven at 60 °C for 48 h. Ultrathin sections (70 nm) were cut on a Leica EM UC7 ultramicrotome using a diamond knife, placed on grids and stained with heavy-metal uranyl acetate and lead citrate for 8 min each. Imaging was performed on an FEI Tecnai G2 Spirit TEM (FEI Company Hillsboro, OR, USA) at 100 kV at 1400× and 4800× magnification.

### 2.12. Cryogenic Electron Microscopy (Cryo-EM)

Extracellular vesicles isolated from tissue sections and from plasma were vitrified by applying 3 μL of EV isolates to lacey carbon 300 Cu mesh grids, approximate grid hole size 63 microns (PELCO TEM, Quorum Technologies Ltd., Laughton, UK). The grids were glow-discharged at 25 mA for 30 s prior to use (GloQube Plus Glow Discharge System, Quorum Technologies Ltd., Laughton, UK). A Vitrobot IV (Thermo Fisher) was used for vitrification, with the chamber maintained at 100% humidity and 12 °C; following application, samples were equilibrated for 30 s, then blotted for 3 s prior to plunge cooling in liquid ethane. The samples were screened and imaged on a Glacios cryo-TEM (Thermo Fisher Scientific, Waltham, MA, USA) operating at an accelerating voltage of 200 kV, equipped with a zero-loss Selectris energy filter with a slit width of 10 eV and Falcon IV camera operating in counting mode. Images were acquired at 11,500× nominal magnification and defocus −15 microns (Supplementary Figure S2); and 79,000× nominal magnification and defocus of −2 microns with a total dose of ~15–20 e/Å^2^.

### 2.13. Protein Quantification

EZQ protein quantification kit (Thermo Fisher Scientific, Waltham, MA, USA), with ovalbumin standard curve (0–2000 µg/µL), was used to determine the total protein in the samples, as per the manufacturer’s recommendations. Briefly, protein standards, EV-containing samples (diluted 1:2–1:5 in 0.2 µm filtered PBS), and tissue homogenate samples (diluted 1:5–1:50 diluted in 0.2 µm filtered PBS) were spotted onto the assay paper in triplicate, allowed to dry at RT. Protein-spotted assay paper was washed in 40 mL of methanol for 5 min. Protein-spotted assay paper was dried at RT, and proteins were stained in 40 mL of the EZQ protein quantification reagent for 30 min at RT, with gentle agitation. Following staining, assay paper was rinsed for 2 min in rinse buffer (10% methanol, 7% acetic acid), for a total of three times. Assay was analysed using Bio-Rad Gel Doc EZ System (Bio-Rad, Hercules, CA, USA).

### 2.14. Detection and Quantification of EV-Enriched and Contaminant Markers

#### Protein Digestion

Fifty microlitres of EVs (~50 µg of protein) were prepared for targeted proteomic analysis. EVs were lysed by three consecutive freeze-thaw cycles followed by vortex for 10 min at room temperature using a MixMate sample mixer (Eppendorf, Hamburg, Germany). The samples were combined with 25 µL of 0.3% RapiGest surfactant (Waters, Milford, MA, USA) in 50 mM ammonium bicarbonate (pH 7.8) and incubated with dithiothreitol (12.5 mM) for 90 min at 60 °C. After cooling to room temperature, the samples were combined with iodoacetamide (23.5 mM) and incubated in the dark for 60 min in a 37 °C shaking water bath. Trypsin Gold (Promega, Sydney, Australia) was added to the protein samples in a ratio of 1:50 (*w*/*w*) and incubated for 17 h in a 37 °C shaking water bath. Ten microlitres of 10% *v*/*v* formic acid were added and incubated at 37 °C for a further 30 min to terminate digestion and remove Rapigest. The samples were centrifuged at 16,000× *g* for 10 min at 4 °C and clear supernatant was extracted and spiked with stable-isotope-labelled internal standard peptides.

### 2.15. Liquid Chromatography Mass Spectrometry (LCMS)

EV tryptic peptides were injected for analysis by LCMS using methods described in Newman et al., 2022 [22] and Newman et al., 2024 [23] to determine abundance of EV markers, co-isolated contaminants, and target proteins of differentially regulated transcripts. Transcript target proteins were included based on in-house capacity to perform quantitative proteomic analysis and previously reported detection in liver-derived EVs [23]. Peptide sequences are given in Table 2. Chromatographic separation was performed on an Agilent Advance Bio Peptide Map column (100 mm × 2.1 mm, 2.7 µm) using an Agilent 1290 Infinity II liquid chromatography system coupled to an Agilent 6495B Triple Quadrupole Mass spectrometer (Agilent Technologies, Santa Clara, CA, USA) for multiple reaction monitoring. Identity of detected endogenous analytes was confirmed based on retention time and ion transition ratios matching spiked SIL peptides. Peak areas of endogenous analytes were normalised to corresponding SIL peak area, and absolute quantification was performed using standard curves of SIL spiked into EV matrix.

### 2.16. RNA Isolation

Total tissue and EV RNA was isolated using TRIzol LS^TM^ Reagent (Thermo Fisher Scientific, Waltham, MA, USA) using previously published protocol [24]. Briefly, 750 µL TRIzol LS (Thermo Fisher Scientific, Waltham, MA, USA) was added to 200 µL of EVs (EVs isolated from 2mL of plasma) or 200 µL of tissue homogenate (resulting 0.01 g of tissue), 200 mL of chloroform was added and each tube, and the samples were mixed using a MixMate (Eppendorf, Hamburg, Germany) set to 1400 rpm, followed by 15 min centrifugation at 12,000× *g* at 4 °C. Aqueous phase was transferred to a new protein LoBind tube (Eppendorf, Hamburg, Germany), mixed with 500 mL of isopropanol and 1 mL RNase-free glycogen (20 mg/mL) and incubated for 10 min at RT. Samples were centrifuged at 12,000× *g* at 4 °C, and the resulting pellet was resuspended in 1 mL of ice cold 80% ethanol. The samples were centrifuged at 7500× *g* at 4 °C, and the resulting pellet was resuspended in 30 µL of RNase-free water, incubated at 60 °C for 15 min, and stored at −80 °C until required for analysis. Total tissue RNA isolation was performed using the same protocol.

### 2.17. Small RNA Sequencing

Total EV RNA (EVs isolated from 2mL of plasma) was used for sequencing analysis. Extracted RNA was prepared using QIAseq miRNA library kit (Qiagen, Hilden, Germany) and quantified using Qubit 1× dsDNA High Sensitivity assay kit (Invitrogen, Carlsbad, CA, USA). Library-size analysis was performed using QIAxcel high-resolution kit (Qiagen, Hilden, Germany), followed by library conversion and circularisation using MGIEasy Universal Library Conversion kit (Qiagen, Hilden, Germany). DNB generation and sequencing of samples was performed using DNBSEQ-G99RS High-throughput Sequencing Reagent Set (G99 App-C FCL SE100) on MGI DNBSEQ-G99 sequencer (MGI Tech, Shenzhen, China), achieving sequencing depth ranging from 8.6 to 14.4 million inputs per sample. Following sequencing, read quality filtering and mapping were performed. Unique Molecular Identifiers (UMIs) were used to group reads, filter out discarded sequences, and remove duplicates, resulting in high-quality merged UMI reads with average Q scores ranging from 39.37 to 49.62. The reads were subsequently mapped to the Homo sapiens reference genome (miRBase_v22) and the piRNA database (piRNAdb_hsa.v1_7_6). The overall mapping percentage varied by sample, ranging from 13.41% to 79.43%. Following alignment, raw RNA sequencing counts were normalised to Counts per Million (CPM). The raw RNA sequencing reads were deposited in the NCBI Sequence Read Archive (SRA) database (BioProject accession: PRJNA1480382).

### 2.18. Data Analysis

EV characterisation data was analysed using GraphPad Prism (version 10.4) and presented as mean with range in the text and graphed as mean ± SD. Differences between three groups were assessed using nonparametric analysis with Friedman test. Differences between two groups were assessed by Wilcoxon matched-pairs signed rank-test.

Primary bioinformatic processing of the sequencing data, including adapter trimming, read quality filtering, and mapping to the human reference genome, was performed by the sequencing facility using standardised analytical pipeline. High-quality mapped reads were aggregated into a raw count matrix.

Raw RNA sequencing counts were normalised to Counts per Million (CPM) for analyses of transcript abundance, correlations, and data visualisation. A CPM threshold of 5 in at least one biological replicate was applied to minimise noise from low-abundance background signal while retaining RNAs that were reliably detected across biological replicates. Replicates with CPM < 5 were designated CPM = 0, and transcripts with CPM < 5 in all replicates were excluded. This threshold was selected based on density distributions showing distinct separation between background signal and consistently detected transcripts across a broad dynamic range, while avoiding overly stringent filtering that could exclude biologically relevant moderately expressed RNAs. GraphPad Prism (version 10.4) was also used to determine Spearman correlation coefficients and generate the correlation matrix and scatter plots. For correlation analyses, CPM normalised transcript abundance data was log-transformed to ensure data normalisation and improve interpretability.

Differential expression analysis was conducted using the pyDESeq2 package in Python (version 3.12), and adjusted *p* values were corrected for multiple testing using the Benjamini–Hochberg false discovery rate (FDR) method. Significant differentially expressed transcripts had a log fold change > 0.5 and adjusted *p* value < 0.05.

Principal component analysis was performed using ScanPy (version 1.10.4) and data visualisation, including Principal Component Analysis (PCA) plots, heatmaps of similarity matrices, and volcano plots for differential expression, conducted using Seaborn (version 0.13) and Matplotlib (version 3.9) libraries. Proportional Venn diagrams were generated using DeepVenn (https://www.biovenn.nl, accessed 14 January 2025) [25].

piRNA sequencing reads were mapped to their respective National Center for Biotechnology Information (NCBI) name using piRNA database version 1.8.0 (www.pirnadb.org), then piRQuest V.2 [26] was used to identify transcripts reportedly dysregulated in HCC and predicted targets.

Using miRNA Enrichment Analysis and Annotation tool (miEAA) [27], over-representation analysis was performed on the significant differentially expressed miRNA transcripts in plasma EVs at timepoints 1 and 2 and cross-referenced to the Kyoto Encyclopaedia of Genes and Genomes (KEGG) and Gene Ontology (GO) databases.

miRTargetLink 2.0 [28] was used to derive a list of predicted protein targets of miRNAs differentially regulated between plasma EV timepoints 1 and 2. For proteins predicted to be regulated by multiple differentially expressed miRNAs, an aggregate normalised to fold change score was calculated to estimate the combined regulatory pressure of differentially expressed transcripts. Each miRNA was assigned a weighted rank score incorporating both the magnitude of differential expression and statistical confidence. Specifically, transcripts with larger log fold changes and lower adjusted *p*-values were assigned higher ranks according to the following equation [24]:

rank = −log10(log2FC × adjusted p-value)

Then, individual transcript fold changes were weighted according to their calculated rank scores using the following equation:

$$Normalised\;FC\;aggregate=\frac{\sum_{i=1}^{n}\left({rank}_{i}\times {FC}_{i}\right)}{\sum_{i=1}^{n}\left({rank}_{i}\right)}$$

where *FC_i_* represents the fold change of each transcript and *rank_i_* represents the corresponding weighted rank score. This weighted aggregation approach reduces disproportionate influence from weakly altered or low-confidence transcripts while emphasising miRNAs with stronger evidence of differential expression. The fold change value was then used in the exploratory correlation analysis with abundance of respective protein targets in the plasma EVs.

### 2.19. Transparent Reporting

**Figure 1:**
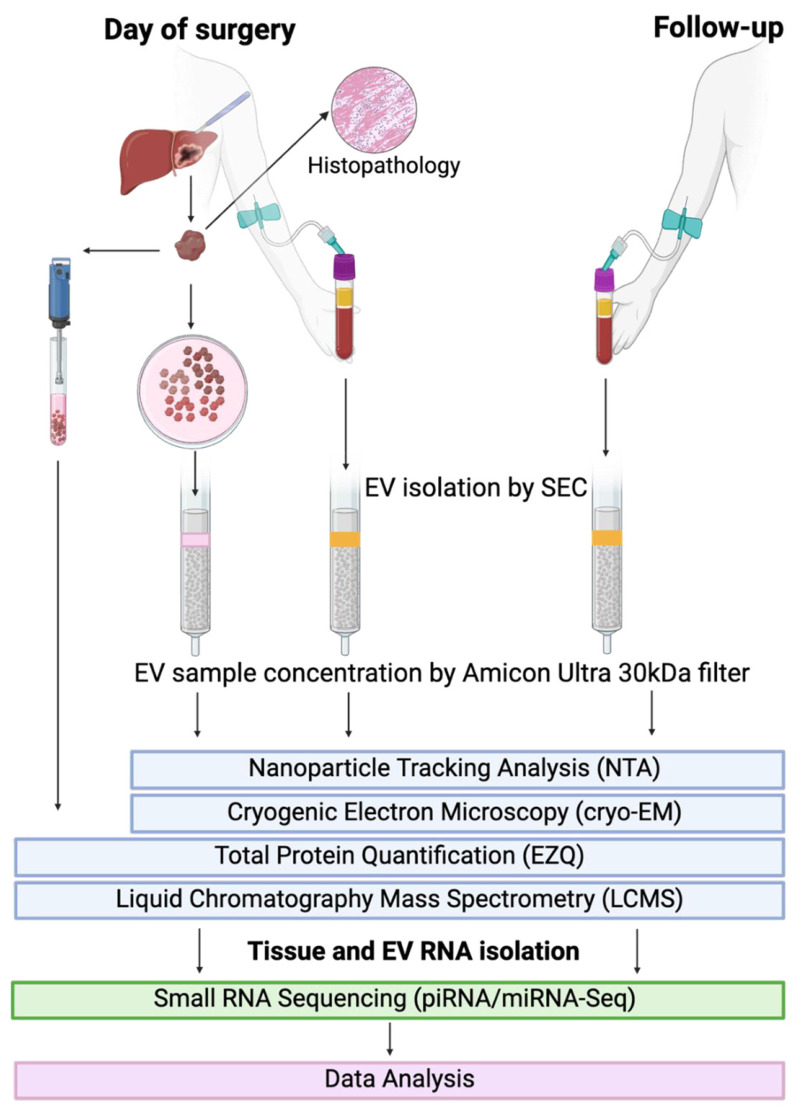
Study design. Liver tissue and blood was collected at time of liver resection surgery and blood was collected at follow-up. EVs were isolated by size exclusion chromatography from plasma and tissue and RNA extracted for small RNA sequencing.

Experimental details were submitted to EV-TRACK platform to facilitate transparency, interpretation, and reproduction of this study. EV_TRACK ID: EV250025. Study design is shown in Figure 1.

While the molecular focus of this study is on HCC, the initial EV characterisation including size distribution, particle yield, and classical EV marker expression was conducted on a mixed clinical cohort encompassing both primary HCC and CRLM. This combined approach was used to determine whether global EV populations exhibit comparable properties across different hepatic malignancies at the time and post–surgical resection. However, recognising that primary and metastatic liver cancers possess fundamentally different biological and immune microenvironments, all subsequent proof-of-concept transcriptomic analyses were strictly restricted to the HCC cohort to eliminate disease heterogeneity as a confounding variable.

## 3. Results

### 3.1. Purity Assessment and Characterisation of EVs Isolated from Solid Tissue and Plasma Samples

Tumour tissue and plasma were collected from a mixed cohort of patients with liver cancer (*n* = 7), including primary hepatocellular carcinoma (HCC) and colorectal cancer patients with liver metastases (CRLM) (Table 1). This combined cohort was used for initial EV characterisation to determine if global EV populations follow similar baseline trends in particle number, size, and standard EV markers (CD9, CD81, TSG101) across different liver malignancies. However, acknowledging the markedly different biological and immune microenvironments of these distinct disease entities, subsequent proof-of-concept transcriptomic analyses were restricted to the HCC cohort to avoid confounding variables.

**Figure 2:**
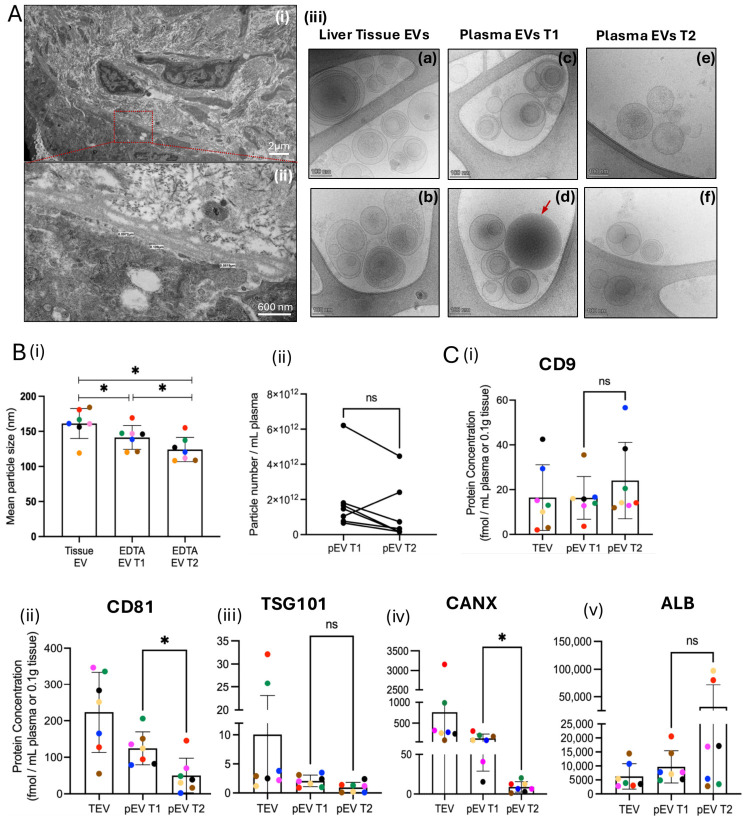
Purity assessment and characterisation of extracellular vesicles isolated from liver tissue (TEV) and plasma (pEV) of patients with primary and metastatic liver cancer at time of surgery (T1) and follow-up (T2), *n* = 7 biological replicates. (**A**) (**i**,**ii**) Transmission electron microscopy images of liver sections and (**iii**) cryogenic electronic microscopy images of isolated EVs: (**a**,**b**) liver tissue EVs, (**c**,**d**) plasma EVs isolated at (T1), (**e**,**f**) plasma EVs isolated at (T2); (**B**) Nanoparticle tracking analysis showing (**i**) mean particle size (nm) and (**ii**) particle number normalised to plasma volume. (**C**) Targeted liquid chromatography mass spectrometry quantitation of EV marker proteins and contaminants: (**i**) CD9, (**ii**) CD81, (**iii**) TSG101, (**iv**) Calnexin (CANX) and (**v**) Ablumin (ALB). Concentration of protein analytes is normalised to starting material (i.e., 0.1 g of tissue or 1 mL of plasma). Data presented as mean ± SD. Statistical testing was performed using Wilcoxon matched-pairs signed-rank test. * *p* < 0.05, ns: not significant.

Resected tumour tissue sections were imaged by TEM to visualise cellular architecture and the presence of vesicles. Particles exhibited size, morphology, and staining characteristic to EVs and were observed in the extracellular space between intact hepatic cells (Figure 2A(i,ii)). Cryo-EM was used to image EV samples and to observe their morphology. Extracellular vesicles isolated from resected tumour tissue were large, multilamellar 80 to 410 nm in size (Figure 2A(a,b)). Similarly to tissue EVs, plasma EVs isolated on the day of surgery (T1) were multilamellar and similar in size and appearance to tissue EVs and ranged between 70 to 305 nm in size (Figure 2A(c,d)). Non-EV contamination of 280 nm was observed (Figure 2A(d)). Plasma EVs isolated from bloods collected at follow-up appointment (T2) were predominantly single membrane bound with less than half vesicles being multilamellar, ranging 80 to 190 nm (Figure 2A(e,f)).

Nanoparticle tracking analysis (NTA) was used to determine the size and concentration of particles in EV isolates (Figure 2B(i)). Particle concentration data are reported as total yield per 400 µL of EVs isolated from 0.1 g of liver tissue; plasma particle concentration is reported as total yield per 1mL of plasma. Following the same trend observed by electron microscopy, particles isolated from resected tissue were significantly larger compared to particles isolated blood on the day of surgery (T1; *p* < 0.05), and follow-up bloods (T2; *p* < 0.01) and had mean (± range) particle sizes of 161.3 (119.1–184.2), 141.3 (120.2–169.2), and 124.1 (108.3–155.1) nm, respectively. The mean (±range) particle concentration for EVs isolated from resected tissue was 5.59 × 10^11^ (1.09 × 10^11^–1.87 × 10^12^). There was no significant difference (*p* = 0.15) in particle concentration between blood EV samples at T1 and T2, 1.94 × 10^12^ (6.66 × 10^11^–6.21 × 10^12^), and 1.21 × 10^12^ (1.61 × 10^11^–4.46 × 10^12^) particles/mL, respectively (Figure 2B(ii)).

Targeted Liquid Chromatography Mass Spectrometry (LCMS) was used to assess the abundance of EV markers and contaminant protein in liver tissue EVs and matched EVs isolated from plasma (Figure 2C). Liver tissue EV data is presented for comparison only and was not included in statistical analysis. There was no significant difference in classical EV markers CD9 (*p* = 0.37) and Tumour Susceptibility Gene 101 (TSG101) abundance (*p* = 0.15), while CD81 was significantly higher in pEVT1 compared to pEVT2 (*p* < 0.05). Calnexin (CANX) is classically considered an endoplasmic reticulum contaminant; however, recent evidence indicates it may be inherently present in certain EV subtypes, including large EVs [29]. Calnexin was significantly enriched in pEVT2 EVs (*p* < 0.05), which may reflect shifts in EV biogenesis or the secretion of specific vesicle subpopulations rather than strictly intracellular contamination. Lastly, albumin was assessed as a marker of soluble protein contamination. Albumin abundance was highly variable in pEVT2 and on average was 3.5-fold higher in pEVT2 than pEVT1 but did not reach statistical significance (*p* = 0.29).

### 3.2. Abundance and Profile of Transcripts in Plasma EVs and Liver Tissue

**Figure 3:**
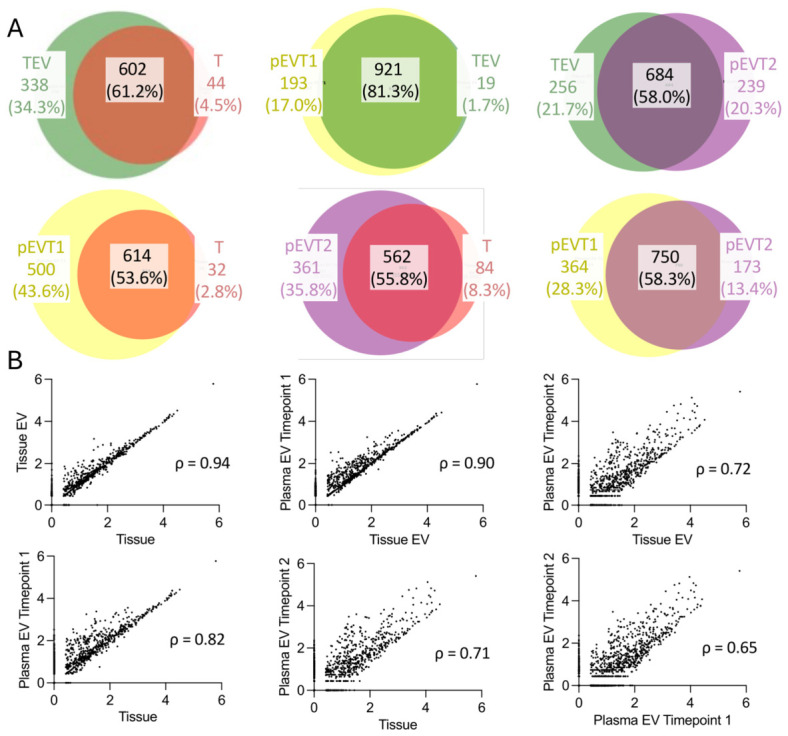
Small RNA sequencing of liver tissue (T), EVs isolated from liver tissue (TEV) and plasma at time of surgery (pEVT1) and follow-up (pEVT2). (**A**) Abundance of unique and shared transcripts detected between sample types. Proportional Venn diagrams generated using DeepVenn. (**B**) Correlations of transcript abundance between sample types using mean counts per million (CPN) values from *n* = 3 biological replicates, calculated by Spearman ρ.

Small RNA sequencing was performed on liver tissue (T) and EVs derived from liver tissue (TEV) and plasma EVs collected at the time of resection (pEVT1) and follow-up (pEVT2) at least three weeks later. Transcriptomic analyses were limited to the subset of patients with primary liver cancer (*n* = 3). The majority of transcripts detected in liver tissue (61.2%) were also identified in TEV, with 34.3% of transcripts unique to TEV (Figure 3A).

Notably, the profile of plasma EVs at timepoint 1 (pEVT1) showed substantial overlap of 81.3% with TEV transcripts, which was markedly reduced at timepoint 2 (pEVT2, 58%). Despite this, the overlap between plasma EVs and liver tissue transcripts remained consistent across timepoints, in each case > 53% were present in both sample types. At timepoint 2, 173 transcripts were uniquely detected in plasma EVs that were absent at timepoint 1, highlighting the temporal changes in plasma EV transcript profiles following surgery.

To determine the relationship of transcript abundance between sample types, mean counts per million (*n* = 3 biological replicates) of each transcript were plotted and Spearman correlations calculated (Figure 3B). TEV and pEVT1 each correlated strongly with the liver tissue (ρ = 0.94 and ρ = 0.82, respectively). Meanwhile, plasma EVs at timepoint 2 exhibited moderate correlations with liver tissue (ρ = 0.65–0.72), reflecting divergence in the RNA profiles over time. Together, these data suggest that the plasma EVs exhibit temporal divergence in transcript composition.

### 3.3. Differential Expression Analysis of Liver and Plasma EV Small RNA Transcriptome

**Figure 4:**
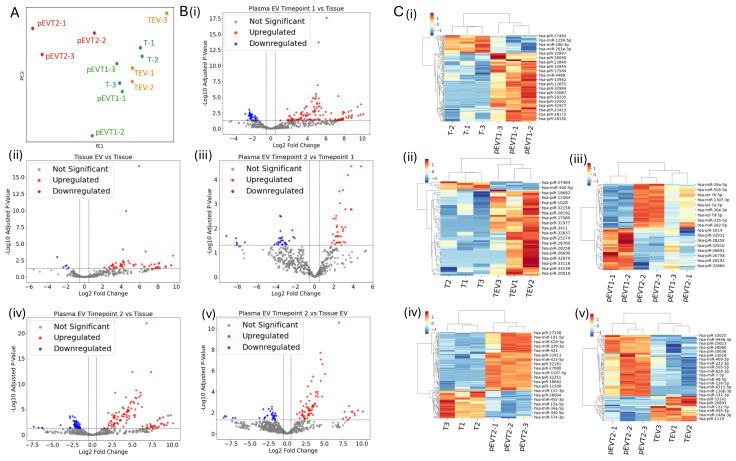
Differential expression analysis of small RNA profiles. (**A**) Principal Component Analysis (PCA) of tissue, tissue EVs and plasma EVs. (**B**) Volcano plot comparisons of (**i**) plasma EVs at T1 and liver tissue (**ii**) Liver tissue EVs and corresponding liver tissue, (**iii**) plasma EVs at T1 and T2, (**iv**) plasma EVs at T2 and liver tissue, (**v**) plasma EVs at T2 and liver tissue EVs; (**C**) Hierarchical cluster maps of differentially regulated transcripts between each sample type: (**i**) liver tissue and plasma EVs at T1, (**ii**) liver tissue and liver tissue EVs, (**iii**) plasma EVs at T1 and T2, (**iv**) liver tissue EVs and plasma EVs at T2, and (**v**) liver tissue and plasma EVs at T2. Differential expression analyses were performed using pyDESeq with Benjamini–Hochberg false discovery rate correction.

Differential expression analysis was used to identify transcripts that were relatively enriched or depleted between sample types (Table S1). In line with the previous observation of temporal changes in transcriptomic profile in plasma EVs, the differentially expressed transcripts demonstrated trends toward separation on principal component analysis of pEVT2 samples from the liver tissue, tissue EV, and plasma EVs collected on the same day (Figure 4A, with enlarged PCA and Volano plots provided in Supplementary Figure S3).

A total of 126 transcripts were enriched in plasma EV (T1) relative to the tissue at time of surgery, while 32 were relatively depleted (Figure 4B). There were 51 enriched and 5 depleted transcripts in tissue EVs relative to the tissue, whereas, for TEV and T1 plasma EV, many transcripts exhibited log fold changes > 0.5, though none reached significance. At follow-up, plasma EVs were enriched in 77 transcripts and depleted in 32 transcripts relative to tissue EVs and had 105 enriched and 51 depleted transcripts relative to total tissue. Finally, between the two plasma EV timepoints, there were 33 transcripts relatively enriched at baseline and 35 enriched at follow-up. Hierarchical clustering analysis (Figure 4C, with enlarged heatmaps provided in Supplementary Figure S4) showed consistent clustering of biological replicates by sample type for each of the comparisons, apart from the plasma EV baseline and follow-up samples. Two of three biological replicates at each timepoint clustered together, while pEVT1-3 and pEVT2-1 exhibited different expression profiles from their respective groups, suggesting greater interindividual variability in plasma transcriptomic EV profiles. However, given the limited sample size, these findings should be interpreted to provide exploratory insight into potential transcriptomic shifts associated with surgery and post-operative recovery.

### 3.4. Plasma EVs Reflect Temporal Changes in Biological Pathways at Transcript and Protein Level

Given the potential to use repeated sampling of plasma EVs to longitudinally assess changes in transcriptomic profiles following liver resection, we further investigated the profile of differentially regulated RNA at follow-up. Of the 35 transcripts relatively enriched at timepoint 2, the majority were microRNA species. These upregulated miRNAs were submitted to over-representation analysis using the miEAA online tool, identifying the GO terms and KEGG pathways associated with the predicted target genes of the miRNA transcripts relatively enriched at follow-up (Table S2). Top GO terms significantly enriched include regulation of somatic stem cell population maintenance, fibroblast apoptotic processes, galactose binding, and plasma membrane raft organisation. Meanwhile, significantly enriched KEGG pathways included hedgehog signalling, cytosolic Deoxyribonucleic Acid (DNA)—sensing, and vitamin digestion and absorption.

Of the 33 transcripts relatively depleted at follow-up, just two were microRNA species and the remainders were piwi-interacting RNAs. Two piRNAs, hsa-piR-25038 and hsa-piR-25627, detected in our dataset were enriched at timepoint 1, in line with their reported upregulation associated with HCC. Higher relative abundance of these transcripts may be associated with reduced expression of their respective mRNA targets, diacylglycerol acetyltransferase 2 (DGAT2) and CD5L, in HCC tumours.

**Figure 5:**
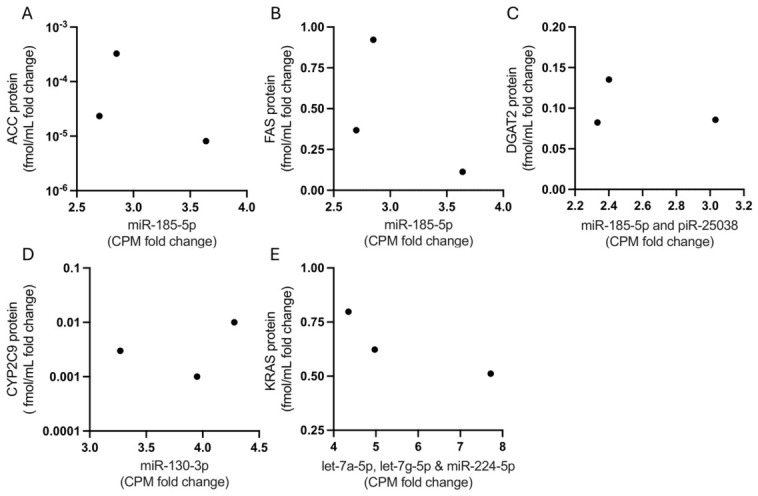
Relationship between differential transcript expression and abundance of predicted protein targets in plasma EVs collected at follow-up (T2) relative to time of surgery (T1) in HCC patients (*n* = 3 biological replicates). Fold changes of transcript counts per million (CPM) were derived from pyDESeq2 differential expression analysis of small RNAseq data, and protein abundance was determined by targeted LCMS analysis in matched plasma EV samples: (**A**) ACC, (**B**) FAS, (**C**) DGAT2, (**D**) CYP2C9 and (**E**) KRAS protein. For proteins predicted to be regulated multiple transcripts, CPM fold changes were normalised using the ranking approach described in Section 2.

The miRTargetLink 2.0 online tool was used to identify mRNA targets of the transcripts differentially expressed at follow-up. Targets were selected for analysis on the protein level by quantitative LCMS, including cytochrome P450 (CYP) 2C9 [miR-130b-3p], sterol regulatory binding protein (SREBF1) [miR-185-5p], which controls expression of several lipogenic enzymes in the liver, such as acetyl Co-A carboxylase (ACC), DGAT2 and fatty acid synthase (FAS), and the KRAS oncogene [let-7a-5p, let-7g-5p, miR-224-5p]. Fold changes in miRNA expression were plotted against that of the target protein abundance in plasma EVs isolated at timepoints 1 and 2 (Figure 5). As described above, DGAT2 protein expression may also be influenced by increased piR-25038. Similarly, two enriched miRNA (hsa-let-7a-5p and hsa-let-7g-5p) and one depleted miRNA (hsa-miR-224-5p) were all predicted to target KRAS. Thus, transcripts were ranked using their log fold change and *p*-value and used to normalise miRNA expression fold changes, as described in the methods. Interestingly, plasma EVs at follow-up exhibited lower levels of target proteins ACC and FAS, corresponding to increased expression of miR-185-5p. DGAT2 protein abundance was also decreased in accordance with changes in expression of miR-185-5p and piR-25038. Similarly, CYP2C9 and Kirsten Rat Sarcoma Viral Oncogene Homologue (KRAS) proteins were each reduced at the protein level in EVs at follow-up, corresponding with relative enrichment-predicted regulatory transcripts.

## 4. Discussion

In this study, transcriptomic profiling was performed on EVs isolated directly from resected HCC tumour tissue and circulating EVs collected both at the time of surgery and at a follow-up interval. This multi-source, longitudinal approach allowed us to assess the overlap in EV transcriptomic profiles and examine dynamic changes in circulating EV content over time. We found that circulating EVs at the time of surgery closely resemble the transcriptomic profiles of both liver tissue and tissue-derived EVs, highlighting the potential of plasma EVs as surrogates for tumour biology. Temporal changes in plasma EV transcriptomic profiles at follow-up suggest a potential avenue to monitor longitudinal molecular following surgery. However, larger prospective studies with clinical outcome data will be required to determine utility for residual disease detection or recurrence surveillance.

Tissue-derived EVs represent a concentrated snapshot of EV composition at the point of resection, providing a useful comparator for both matched tumour tissue and circulating EVs. Unlike conditioned media-derived EVs, tissue EVs reflect the microenvironment in situ, capturing both tumour-intrinsic signals and immediate cross-talk with the surrounding tissue. Optimised protocols for gentle EV isolation from tissue sections, alongside imaging-based confirmation of EV presence in extracellular spaces, ensured we captured biologically relevant vesicles while avoiding contamination from intracellular debris. TEM and cryo-EM analyses confirmed that EVs isolated from blood at surgery were morphologically similar to liver tissue EVs, being predominantly multilamellar. Interestingly, follow-up plasma EVs were smaller and more often single-membrane bound, which may reflect a shift in EV biogenesis or cellular origins post-surgery. The sizing of TEV and pEV by cryo-EM somewhat disagreed with NTA data. Since vesicles are imaged under ultra-low temperature (<−175 °C) by cryo-EM, they are preserved in their native hydrated state and might appear larger when compared to orthogonal methods [30]. Cancer cells have been documented to secrete EVs in larger amounts than normal cells, and these EVs are often larger in size due to the increased shedding rate associated with malignant tumours [31,32].

Despite no significant change in overall EV concentration after surgery, follow-up EVs showed altered molecular cargo, including lower CD81 expression and reduced levels of the ER marker CANX. This aligns with our previous findings that liver tissue EVs are enriched in CANX and other liver-enriched proteins, reflecting tissue of origin [21,23]. While SEC effectively separates EVs from the bulk of soluble proteins, EV purity remains a complex challenge in liquid biopsy applications. We observed variable and occasionally elevated albumin levels in follow-up plasma EVs, highlighting the potential for co-isolation of protein aggregates. Furthermore, a limitation of our current targeted LC-MS panel is the absence of apolipoprotein markers (e.g., ApoA1, ApoB). Consequently, the extent of lipoprotein could not be definitively quantified in this cohort. Future longitudinal studies should incorporate expanded multiplex panel to thoroughly profile lipoprotein contamination alongside classical EV markers. We observed substantial overlap (>80%) in the profile of transcripts in pEVT1 with TEV and a strong correlation (ρ = 0.90) in the abundance of transcripts between the sample types. While the diseased liver is expected to secrete more EVs into the blood, the typical proportion of total circulating EVs in HCC patients is not well established. However, liver-derived EVs were estimated to constitute up to 20% in the plasma from a cohort of patients with non-alcoholic steatohepatitis [33], and another study proposed that numbers of circulating microparticles positive for HepPar1 may serve as an early marker of HCC recurrence [34]. Though a similar proportion of transcripts overlapped between tissue and plasma EVs at each timepoint, the profile in follow-up plasma EVs diverged from that of TEV and T1. Vesicles from various sources, especially immune-derived, entering the tissue from the circulation could contribute to the high concordance of TEV with plasma EV profile at T1. Meanwhile the dynamic shift in plasma EV composition after surgery may reflect both loss of tumour-derived EVs and a compensatory secretion from non-tumour liver tissue and immune cells involved in post-operative repair and inflammation [19,35]. Meanwhile, the changes after surgery reflect a complex interplay between the loss of tumour-derived EVs and a compensatory secretion from non-tumour liver tissue and immune cells involved in post-operative repair and inflammation. It is highly likely that a substantial proportion of the transcriptomic changes observed in the follow-up plasma EVs are driven by this systemic inflammatory response and wound healing, rather than solely reflecting the clearance of tumour-specific biology.

Importantly, the inherent heterogeneity of plasma EVs must be considered when interpreting plasma-circulating signals. Blood plasma is a highly complex biofluid where tumour-derived EVs likely constitute a minor fraction of the total vesicle population, which is otherwise dominated by EVs shed from platelets, erythrocytes, and circulating leukocytes. High background of non-malignant-cell EVs can dilute tumour-specific transcriptomic signatures and introduce systemic “noise” Consequently, the temporal RNA shifts observed in our plasma EV cohorts represent a composite signal. While this composite nature is useful for capturing broad, systemic physiological responses to surgery, it simultaneously complicates the ability to definitively attribute specific transcriptomic alterations exclusively to the tumour or the immediate hepatic microenvironment.

Transcriptomic profiling revealed differential expression of numerous small RNAs across sample types and timepoints. Differences between matched tissue and tissue EVs likely reflect selective RNA packaging into EVs [36], whereas longitudinal changes in plasma EV may suggest evolving molecular processes during post-surgical recovery. Pathway enrichment analysis of predicted targets of miRNAs enriched in follow-up plasma EVs identified associations with immune surveillance (cytosolic DNA sensing, IL-17 signalling), metabolic processes (vitamin digestion and absorption, thiamine metabolism, sulfur metabolism), and liver regeneration and disease progression (Hedgehog signalling, NAFLD). These processes may reflect the physiological responses expected to follow HCC resection, such as tissue repair, systemic metabolic reprogramming, and immune activation. However, these pathway analyses are hypothesis-generating, and further functional studies are required to validate any underlying biological mechanisms. While these data suggest that circulating EV transcriptomic profiling might capture a molecular fingerprint of post-surgical adaptation, any potential implications for recurrence monitoring and risk stratification remain speculative and strictly require future longitudinal clinical validation with documented recurrence outcomes. Distinguishing markers of minimal residual disease from the overwhelming transcriptomic “noise” generated by post-surgical inflammation remains a significant challenge. The enrichment of immune signalling pathways at follow-up underscores that plasma EVs act as a real-time readout of systemic recovery, meaning future biomarker discovery efforts must carefully differentiate between transient inflammatory signatures and true longitudinal tumour dynamics.

In addition to RNA content, circulating EVs carry proteins with potential biomarker value. The composition of EV proteins may reflect both intracellular protein abundance and the transcriptomic state of the originating cells, including selective cargo-loading process and post-transcriptional regulation [37]. Previous work by our group established quantitative LCMS methods for profiling key liver-derived proteins in EVs, including those involved in drug metabolism and chronic liver disease [23]. Notably, several of these proteins are encoded by genes predicted to be targeted by the ncRNAs altered in this study. Although we observed a reduction in protein abundance in follow-up EVs, corresponding to relative enrichment of predicted regulatory transcripts, the small sample size precluded meaningful correlation analysis. Nonetheless, this preliminary observation highlights the potential of circulating EVs to reflect the coordinated regulation of transcript and protein levels and warrants additional multi-omics studies to clarify the utility for tumour monitoring.

The major limitation of this pilot study is small and heterogeneous cohort size. With transcriptomic analyses restricted to three HCC patients, this highly preliminary dataset limits statistical power, the assessment of interpatient variability, and the generalisability of our findings. However, it is important to note that the primary aim of this study was not to perform cross-sectional comparisons between patients, but rather to utilise each patient as their own internal control. By focusing on matched longitudinal sampling, comparing the circulating EV profile at diagnosis to the local tumour tissue, and subsequently tracking the divergence of these markers in the same patient following resection, this proof-of-concept study was specifically designed to capture real-time, intra-patient molecular shifts. While EVs were collected from patients with liver metastases as well as primary liver cancer, revealing a similar trend in the characteristics of tumour derived and plasma EVs at time of surgery and shifting profile at follow-up, acknowledging the markedly different biological and immune microenvironments of these distinct disease entities, subsequent proof-of-concept transcriptomic analyses were restricted to the HCC cohort to avoid confounding variables.

Furthermore, the variability in our follow-up collection intervals (spanning 3 to 7 weeks post-operatively) introduces additional biological complexity. This timeframe encompasses highly dynamic and variable physiological processes, including acute inflammatory responses, wound healing, and active liver regeneration, all of which heavily influence systemic EV secretion and transcriptomic cargo. This temporal variability compounds other inherent patient-specific differences, such as age, baseline liver health, and medication use. However, despite these differing post-surgical timelines and individual clinical variables, the transcriptomic profiles of the follow-up plasma EVs followed a consistent trend of divergence from the baseline tumour signature across all observed patients.

Future studies could explore trends in EV composition beyond this initial recovery period to investigate early signs of recurrence. Lastly, functional analysis of enriched or depleted piRNAs in plasma EVs at each timepoint was limited, as the knowledgebase for the roles of these RNA species in humans is less developed than that of miRNA. Furthermore, due to the limited yield of clinical material from this small cohort, orthogonal validation (such as RT-qPCR) of the key differentially expressed miRNAs and piRNAs was not feasible. Consequently, the transcriptomic shifts identified by our sequencing analysis lack independent technical validation, reinforcing the necessity for these findings to be confirmed in independent, larger cohorts.

## 5. Conclusions

This study highlights the potential of EV-based transcriptomic profiling to capture tumour-associated signals and longitudinal molecular changes following HCC resection. Our findings suggest that plasma EVs may provide a molecular window into both local hepatic biology and broader systemic responses after surgery, serving as a basis for future hypothesis generation. While these observations support the feasibility of circulating EV profiling as a minimally invasive monitoring approach, future studies incorporating larger cohorts, longer term follow-up, and integrated multiomic analysis of EV RNA, protein, and other molecular cargo will be important to define their utility for recurrence monitoring and risk stratification in liver cancer.

## Figures and Tables

**Table 1:** Characteristics of sample population. HCC: hepatocellular carcinoma. CLRM: colorectal cancer with liver metastases.

Demographic	Females	Males
**Histopathology—HCC**	*n* = 0	*n* = 3
Donor age range (years)	-	49–68
Donor age median (years)	-	59
Body Mass Index (BMI; range)	-	23.7–35.7
Body Mass Index (BMI; median)	-	28.2
Cirrhosis	-	2
**Histopathology—CRLM**	*n* = 2	*n* = 2
Donor age range (years)	45–65	71–80
Donor age median (years)	55	75.5
Body Mass Index (BMI; range)	24.8–28.7	26.9–29.1
Body Mass Index (BMI; median)	55	75.5
Cirrhosis	-	-

**Table 2:** Peptide sequences for liquid chromatography mass-spectrometry-targeted proteomic assays. Bold^ indicates stable isotope labelled amino acid.

Protein	Peptide Sequence
Cluster of differentiation protein 81 (CD81)	H2N-QFYDQALQQAVVDDDANNA**K^**-OH
	H2N-QFYDQALQQAVVDDDANNAK-OH
Cluster of differentiation protein 9 (CD9)	H2N-DVLETFTV**K^**-OH
	H2N-DVLETFTVK-OH
Tumour suppressor gene 101 (TSG101)	H2N-GVIDLDVFL**K^**-OH
	H2N-GVIDLDVFLK-OH
Calnexin (CANX)	H2N-IVDDWANDGWGL**K^**-OH
	H2N-IVDDWANDGWGLK-OH
Albumin (ALB)	H2N-LVNEVTEFA**K^**-OH
	H2N-LVNEVTEFAK-OH
Acetyl CoA carboxylase 1 (ACC)	H2N-VNNADDFPNLF**R^**-OH
	H2N-VNNADDFPNLFR-OH
Fatty acid synthase (FAS)	H2N-GVDLVLNSLAEE**K^**-OH
	H2N-GVDLVLNSLAEEK-OH
Diacylglycerol acetyltransferase 2 (DGAT2)	H2N-DTIDYLLS**K^**-OH
	H2N-DTIDYLLSK-OH
Cytochrome P450 2C9 (CYP2C9)	H2N-GIFPLAE**R^**-OH
	H2N-GIFPLAER-OH
Kirsten rat sarcoma virus protein wildtype (K-RAS)	H2N-LVVVGAGGVG**K^**-OH
	H2N-LVVVGAGGVGK-OH

## Data Availability

The datasets generated and analysed during the current study are available from the corresponding author upon reasonable request. De-identified patient data will be shared after publication, subject to institutional ethical guidelines and data transfer agreements, to protect patient privacy. The datasets generated and analysed in this study are available in the NCBI Sequence Read Archive (SRA) repository, BioProject accession PRJNA1480382 https://www.ncbi.nlm.nih.gov/bioproject/1480382 (accessed 22 June 2026).

## Supplementary Materials

The following supporting information can be downloaded at: https://www.mdpi.com/article/10.3390/cancers18132109/s1, Figure S1: Representative histopathology images demonstrating H&E—assisted tissue sectioning approach for liver tissue. Dotted lines indicate cutting boundaries of the tissue samples into two morphologically similar sections. Olympus Brightfield BX53 Upright Microscope; Magnification 40×; Figure S2: Representative Cryo-EM images of liver tissue EVs (A,B) and plasma EVs (C–F) at magnification of 11,500× (A,C,E) and 79,000× (B,D,F). No sharpening, normal contrast; Figure S3: Enlarged Principal Component Analysis and Volcano Plots (A) of Differentially Expressed Small RNAs (B); Figure S4: Enlarged Hierarchical Cluster Maps of Differentially Expressed Small RNAs; Table S1: Significant differentially regulated transcripts between each sample type; Table S2: Results of miRNA Enrichment Annotation and Analysis for GO and KEGG.

## Abbreviations

The following abbreviations are used in this manuscript:

EVs	Extracellular Vesicles
RNA	Ribonucleic Acid
HCC	Hepatocellular Carcinoma
MAFLD	Metabolic Associated Fatty Liver Disease
CRC	Colorectal Cancer
C	Celsius
g	g-force
LN2	Liquid Nitrogen
H&E	Hematoxylin and Eosin
KCl	Potassium Chloride
SEC	Size Exclusion Chromatography
LC-MS/MS	Liquid Chromatography with Tandem Mass Spectrometry
NTA	Nanoparticle Tracking Analysis
TEM	Transmission Electron Microscopy
Cryo-EM	Cryo-Electron Microscopy
LCMS	Liquid Chromatography Mass Spectrometry
SD	Standard Deviation
UMI	Unique Molecular Identifiers
CPM	Counts per Million
PCA	Principal Component Analysis
NCBI	National Center for Biotechnology Information
KEGG	Kyoto Encyclopaedia of Genes and Genomes
GO	Gene Ontology
TSG101	Tumour Susceptibility Gene 101
CANX	Calnexin
pEVs	Plasma Extracellular Vesicles
T1	Timepoint 1
T2	Timepoint 2
T	Tissue
TEVs	Tissue Extracellular Vesicles
DNA	Deoxyribonucleic Acid
DGAT2	Diacylglycerol acetyltransferase 2
CYP	Cytochrome
ACC	Co-A- carboxylase
FAS	Fatty acid synthase
KRAS	Kirsten Rat Sarcoma Viral Oncogene Homologue
ANFF	Australian National Fabrication Facility
