# Supplementary figures and images for: Circulating Extracellular Vesicles Reflect Dynamic Shifts in Liver Transcriptome Following Tumour Resection
Source: Cancers (Basel). 2026 Jun 29;18(13):2109. doi: 10.3390/cancers18132109 (PMC13359854; doi:10.3390/cancers18132109)

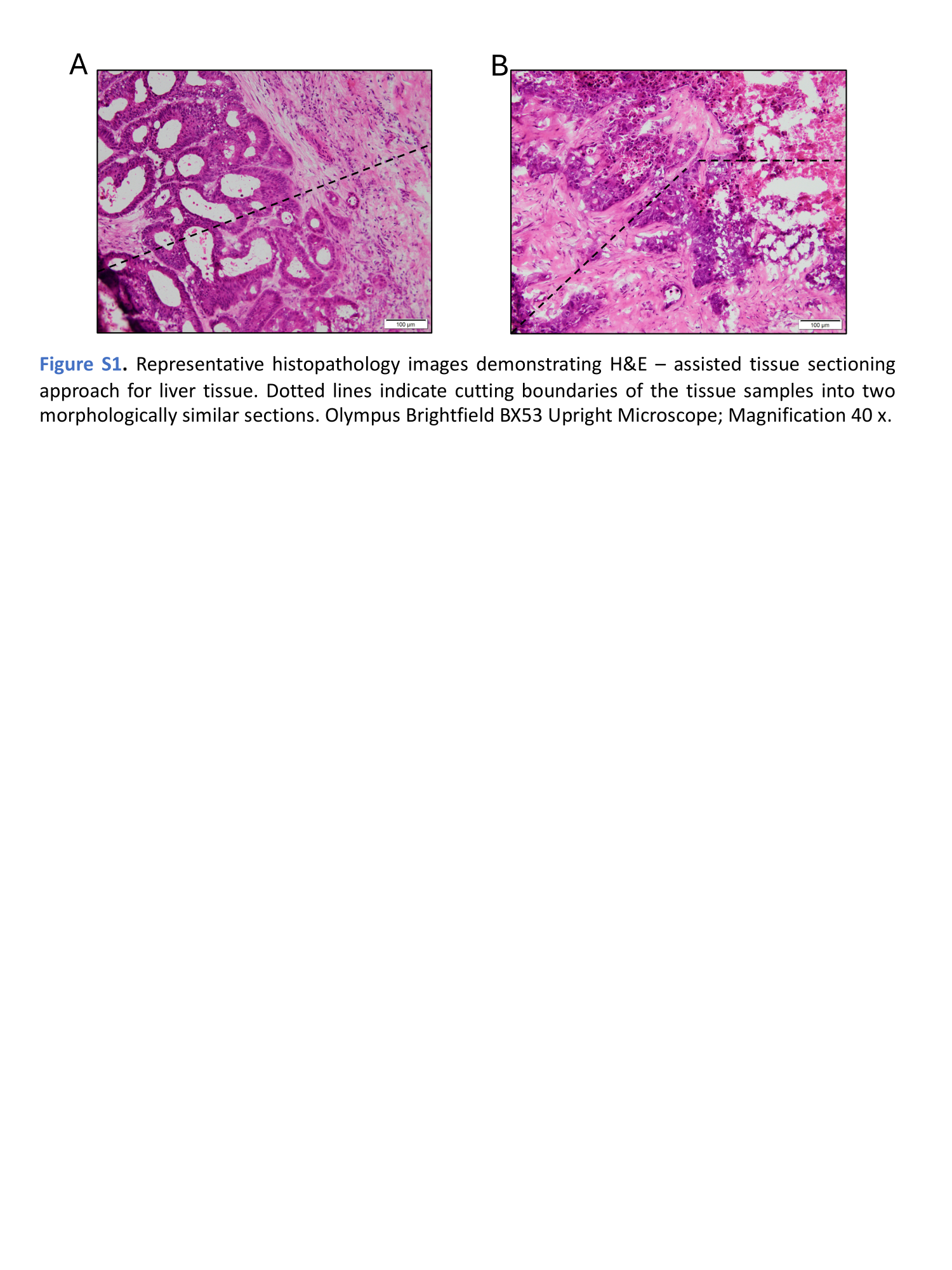

Supplement: Supplementary file 1 [file cancers-18-02109-s001.zip › Supplementary Figure S1.tiff]

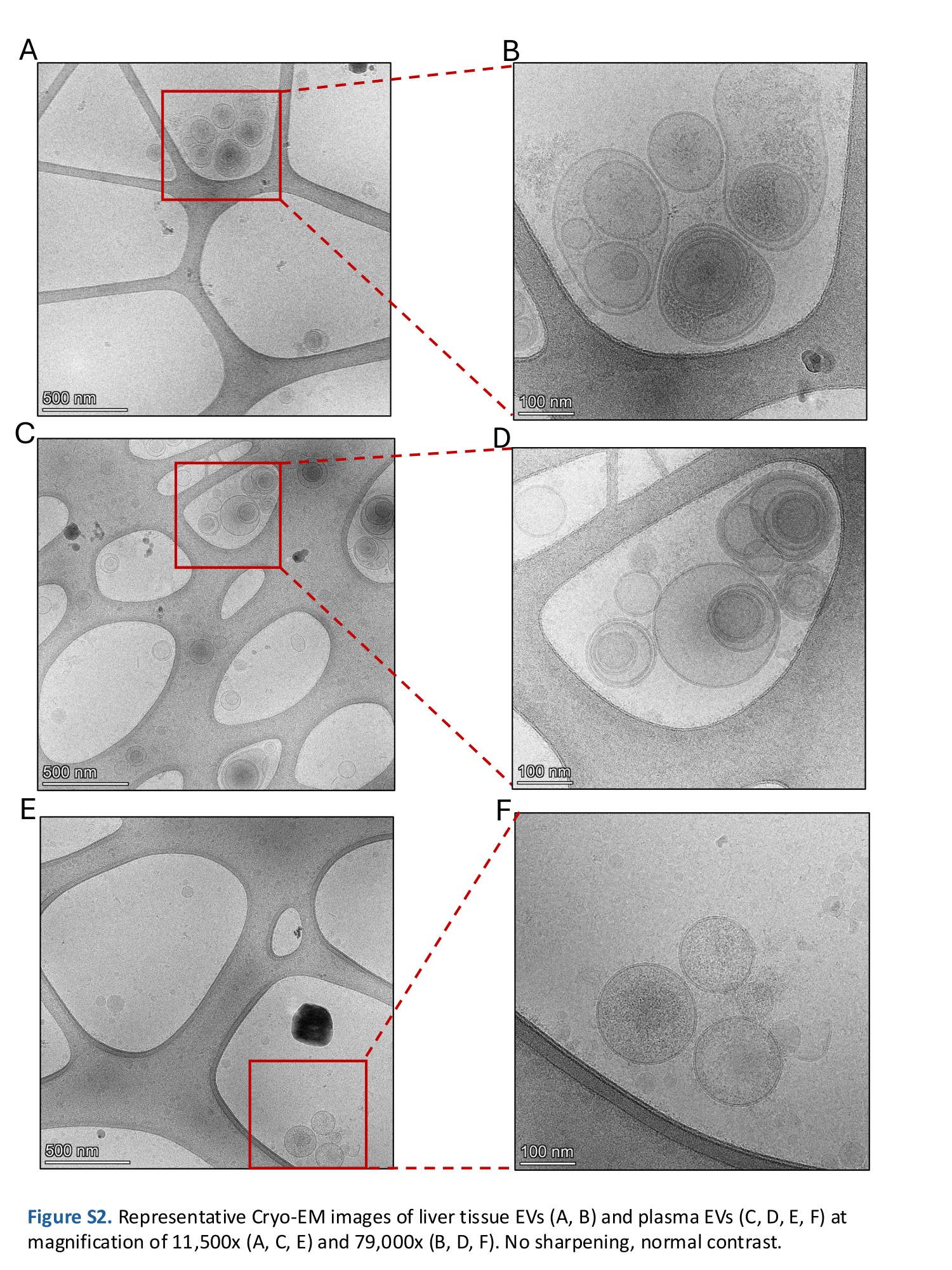

Supplement: Supplementary file 1 [file cancers-18-02109-s001.zip › Supplementary Figure S2.tiff]

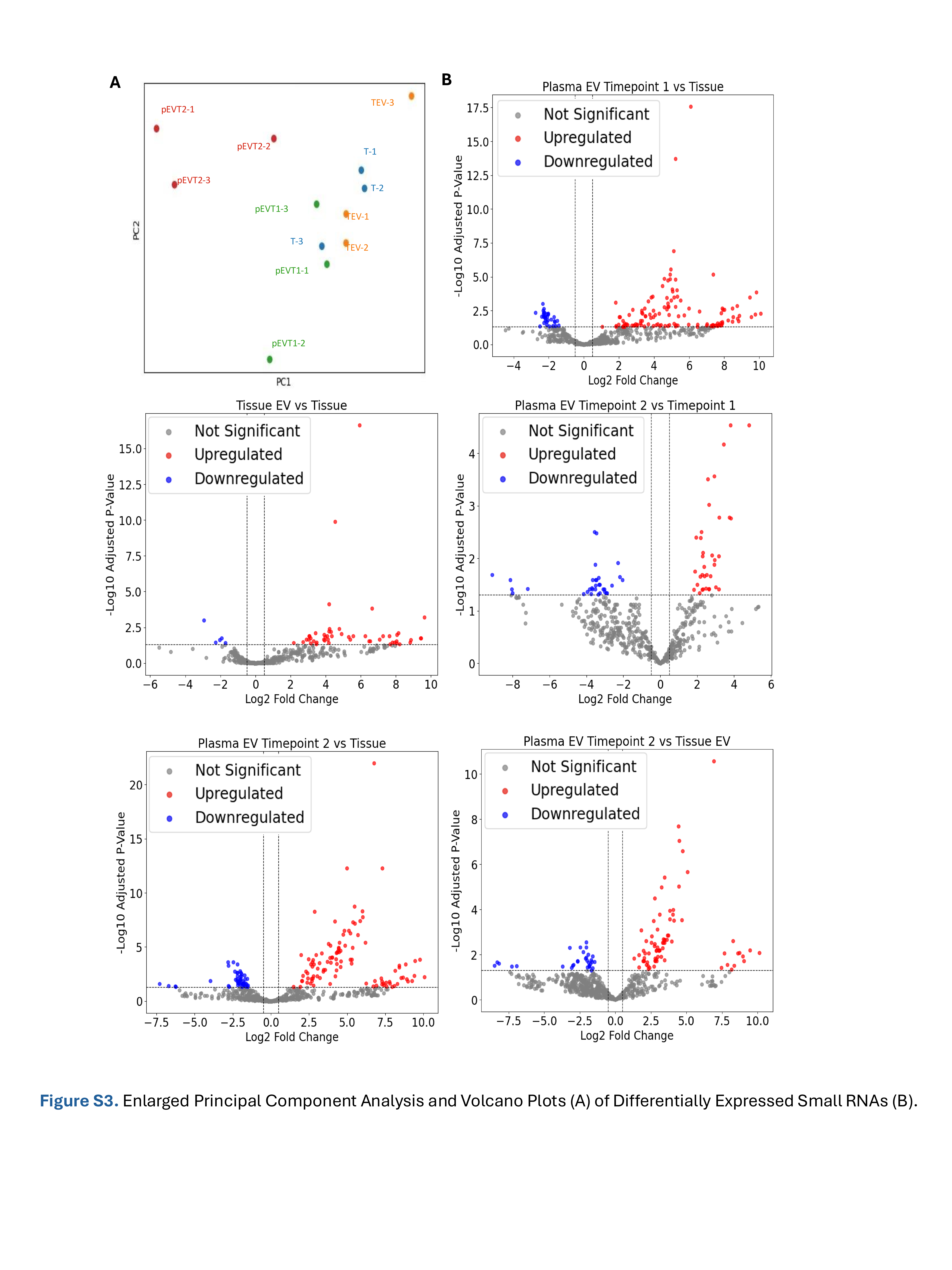

Supplement: Supplementary file 1 [file cancers-18-02109-s001.zip › Supplementary Figure S3.tiff]

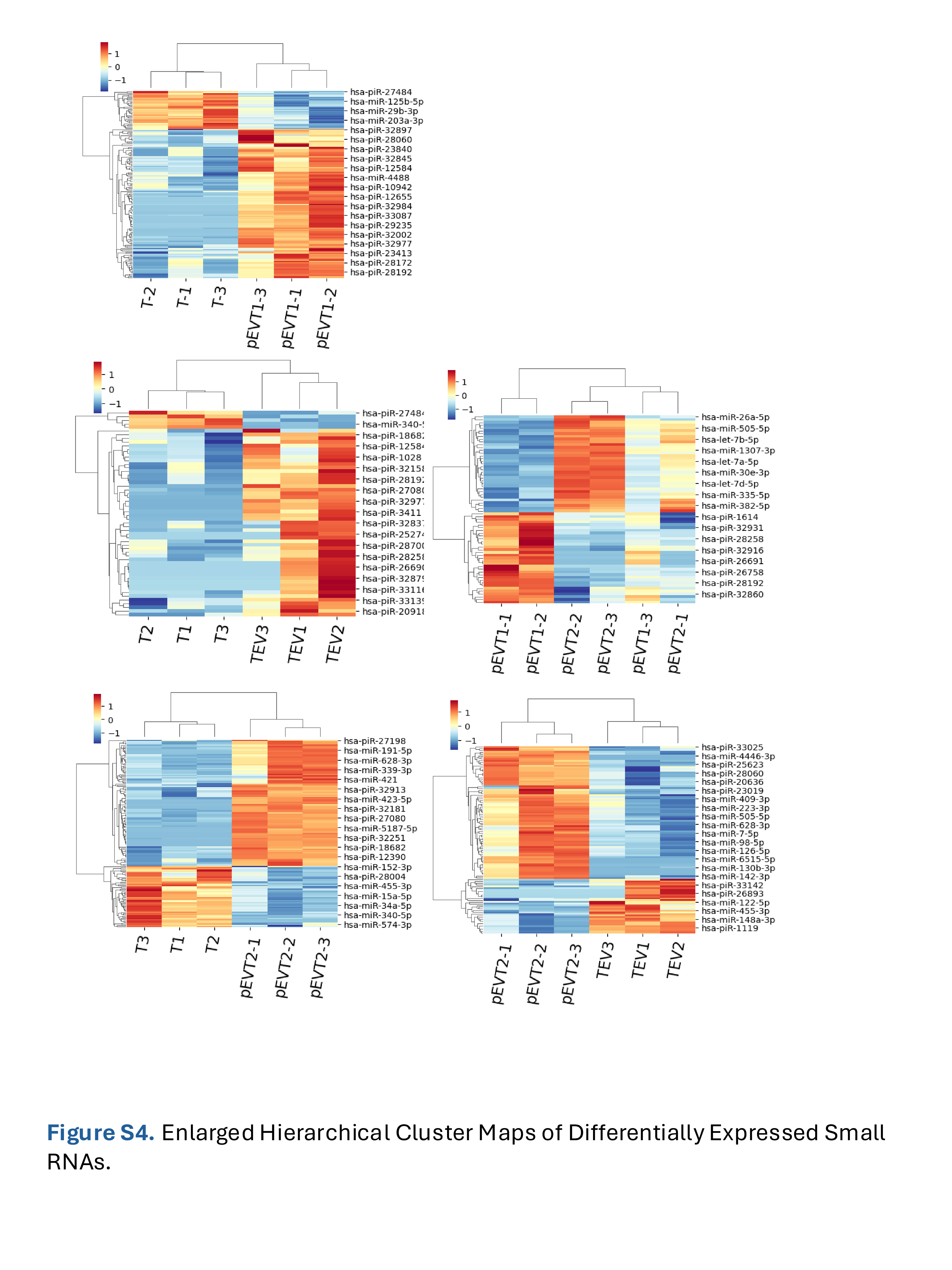

Supplement: Supplementary file 1 [file cancers-18-02109-s001.zip › Supplementary Figure S4.tiff]
